# Optimization and Validation of a Harmonized Protocol for Generating Therapeutic-Grade Dendritic Cells in a Randomized Phase II Clinical Trial, Using Two Varied Antigenic Sources

**DOI:** 10.3390/vaccines12020112

**Published:** 2024-01-23

**Authors:** Abirami Seetharaman, Vasanth Christopher, Hemavathi Dhandapani, Hascitha Jayakumar, Manikandan Dhanushkodi, Narmadha Bhaskaran, Swaminathan Rajaraman, Rama Ranganathan, Shirley Sunder Singh, Varalakshmi Vijayakumar, Arivazhagan Rajamanickam, Anil Suri, Nirmala Jagadish, Thangarajan Rajkumar, Priya Ramanathan

**Affiliations:** 1 Department of Molecular Oncology, Cancer Institute (WIA), Adyar, Chennai 600036, India; abiramiseetharaman@gmail.com (A.S.); biohemavathi@gmail.com (H.D.); jhascitha@gmail.com (H.J.); dmani1982@gmail.com (M.D.); drtrajkumar@gmail.com (T.R.); 2 Department of Radiation Oncology, Cancer Institute (WIA), Adyar, Chennai 600036, India; jcvcmd@gmail.com; 3 Department of Transfusion Medicine, Cancer Institute (WIA), Adyar, Chennai 600036, India; drnarmadha01@gmail.com; 4 Department of Epidemiology, Cancer Institute (WIA), Adyar, Chennai 600036, India; r.swaminathan@cancerinstitutewia.org (S.R.); ramaraghu91@yahoo.com (R.R.); 5 Department of Pathology, Cancer Institute (WIA), Adyar, Chennai 600036, India; drshirleysundersingh@gmail.com; 6 Department of Microbiology, Cancer Institute (WIA), Adyar, Chennai 600036, India; vaaraabi@gmail.com; 7 Department of Clinical Biochemistry, Cancer Institute (WIA), Adyar, Chennai 600036, India; r.arivazhagan1958@gmail.com; 8 National Institute of Immunology, Department of Biotechnology (DBT), Ministry of Science and Technology, New Delhi 110067, India; anilsuri4@gmail.com (A.S.); nirmalajagadish26@gmail.com (N.J.); 9 Centre for Cancer Immunotherapy, Sri Ram Cancer & Superspeciality Centre (SRCC), Mahatma Gandhi Medical College and Hospital, Jaipur 302022, India; 10 Research Oncology, Medgenome, Bangalore 560099, India; 11 IIT Madras, Chennai 600036, India; 12 Department of Nano sciences and Molecular Medicine, AIMS, Kochi 682041, India

**Keywords:** immunotherapy, clinical trial, large-scale dendritic cell production, cervical cancer, cell therapy medicinal products (CTMP)

## Abstract

Autologous dendritic cell (DC)-based immunotherapy is a cell-based advanced therapy medicinal product (ATMP) that was first introduced more than three decades ago. In the current study, our objective was to establish a harmonized protocol using two varied antigenic sources and a good manufacturing practice (GMP)-compliant, manual method for generating clinical-grade DCs at a limited-resource academic setting. After obtaining ethical committee-approved informed consent, the recruited patients underwent leukapheresis, and single-batch DC production was carried out. Using responder-independent flow cytometric assays as quality control (QC) criteria, we propose a differentiation and maturation index (DI and MI, respectively), calculated with the QC cut-off and actual scores of each batch for comparison. Changes during cryopreservation and personnel variation were assessed periodically for up to two to three years. Using our harmonized batch production protocol, the average DI was 1.39 and MI was 1.25. Allogenic responder proliferation was observed in all patients, while IFN-gamma secretion, evaluated using flow cytometry, was detected in 10/36 patients and significantly correlated with CD8+ T cell proliferation (*p* value-0.0002). Tracking the viability and phenotype of cryopreserved MDCs showed a >90% viability for up to three years, while a mature DC phenotype was retained for up to one year. Our results confirm that the manual/semi-automated protocol was simple, consistent, and cost-effective, without the requirement for expensive equipment and without compromising on the quality of the final product.

## 1. Introduction

Dendritic cells are potent antigen-presenting cells expressing ample co-stimulatory molecules (CD80, CD86, and CD40) that are capable of stimulating the innate and adaptive immune systems. These cells can be generated from various progenitors using different cytokine cocktails into immature myeloid DCs (from CD11c^+^/CD123^−^) or plasmacytoid DCs (from CD11c^−^/CD123^+^) [1,2], and can be loaded with a variety of antigens, including peptides, tumor lysates, DNA, or RNA [3,4,5,6], and matured with pro-inflammatory cytokines to trigger Th1 responses. Mature DCs (MDCs) can migrate to the nearest draining lymph node, where they interact with the immune cells to initiate a specific immune response, which is key to initiating productive anti-tumor responses in vivo [7]. Various academic clinical research centers (ACRCs) from around the world have evaluated the efficacy of dendritic cells (DCs) as therapeutic adjuvants. These centers themselves are involved in conducting clinical trials for such cell-based therapies as they have ready access to patients, enabling clinician researchers to be directly involved in the trials [8,9].

In cancers with a proven link to infectious pathogens, the development of immunotherapeutic approaches may be worthwhile in generating potent immune responses that ultimately target the cancer cell. Cervical cancer, the second most common cancer among women in India [10,11,12], is known to be caused by the human papillomavirus (HPV) [13,14]. The standard of care for advanced cervical cancer is concurrent chemoradiotherapy with a probability of relapse in about 28% to 64% of those who receive treatment at advanced stages (Stage II B to IV A) [15,16,17]. Hence, we conducted a pilot study and established the safety and toxicity of autologous tumor lysate-pulsed dendritic cells previously. This study also showed a potent response in one of four patients who received the DCs [4]. We had previously evaluated small-scale DC production in vitro, and compared autologous tumor lysate-primed DCs with recombinant human sperm-associated antigen 9 (rhSPAG9)-primed DCs [3]. However, it was imperative to establish a protocol that could be harmonized for distinctly varied antigenic sources, with the potential to be scaled up for conducting a clinical trial and without cost escalation, to suit low- or middle-income settings like ours. In the current study, our primary objective was to carry out single-batch scaled-up DC production for a Phase II clinical trial setting using two different antigenic sources—autologous whole tumor lysates and commercially synthesized rhSPAG9. Our focus was developing a harmonized approach for DC generation and the evaluation of each batch of DCs for functional and phenotypic characteristics, along with microbiological sterility. The DCs were also cryopreserved and checked for stability, in terms of their viability and phenotype, periodically for up to three years. Our study showed that the entire generation process could be performed with basic clean-room and equipment settings using personnel trained in the workflow to optimize cost and maintain consistency, ideally suited for resource-constrained settings. Since the study is ongoing, the assessment of the response and efficacy of the treatment protocol is beyond the scope of this manuscript.

## 2. Materials and Methods

### 2.1. Study Plan and Patient Recruitment

This Phase II study was a randomized, double-blind, controlled, three-arm trial (*n* = 18 in each arm). Only Stage III B cervical cancer patients (*n* = 54) who were positive for both HPV and SPAG9 were included in this study, which was registered with the Clinical Trials registry of India (CTRI No. CTRI/2016/12/007530). Based on specific inclusion and exclusion criteria (Supplementary Table S1), after receiving informed consent, which was approved by the Institutional Ethics committee as well as the Drug Controller General of India (DCGI) as per the regulatory guidelines, the patients were randomized to one of three arms: arm I—patients received conventional concurrent chemo-radiotherapy plus a placebo (saline) given intradermally; arm II—patients received conventional concurrent chemo-radiotherapy plus TLPDCs (autologous tumor lysate-primed DCs) given intradermally; and arm III—conventional concurrent chemo-radiotherapy plus rSPDCs (DCs primed with recombinant human SPAG9). The patients underwent leukapheresis and DCs were batch-produced as either TLPDCs (autologous tumor lysate-primed DCs) or rSPDCs (DCs primed with recombinant human SPAG9 protein). Eighteen patient samples were used for the batch production of either DCs, which resulted in 36 batch-produced MDCs.

### 2.2. Maintenance of Clean Room for GMP-Grade Production of DCs

As per the World Health Organization (WHO) requirements [18], our clean-room facility features changing rooms and gowning and de-gowning areas (ISO 8), and leads up to an ISO 6 (class-1000) clean room housing a Class IIA biological safety (ISO 5/class-100) cabinet. The hard stainless-steel-walled structure with HEPA filtration systems maintained the air cleanliness levels, allowing a maximum of 1000 particles (size ≥ 0.5 µm) per cubic meter of inside air for the ISO 6 vaccine production facility. This class-1000 cleanroom filtration system provided a filter coverage of 20–30%. A standard air flow rate of 18–32 CFM (cubic feet per minute) per square foot was maintained as required, and this design ensured protection to the finished product. Particle calibrations were performed biannually, which showed that we could maintain a particle count of an average of 670 particles in the class-1000 facility and 6880 particles in the class-10,000 room. A positive pressure of 15 Pa was maintained consistently. The average temperature and humidity limit maintained in the clean-room facility were 23.1 °C ± 2 °C and 55% ± 4, respectively, for 5 years. The facility was sterilized adhering to GMP regulations, and the microbial contamination of the clean-room environment was assessed before and after sterilization. On average, <2 colony forming units (CFUs)/plate was set as the acceptable limit. The surface sampling of the laboratory work benches routinely ensured the sterility of the clean room before DC production. Standard operating protocols (SOPs) for sterilization and material-handling procedures were developed and strictly adhered to. To reduce the potential risk of contamination of cell cultures, and with consideration of the fact that terminally differentiated matured DCs cannot undergo post-production sterilization, all the processes were carried out in a Class IIA Biosafety cabinet (class-100—Thermo Fisher Scientific, Waltham, MA, USA) housed within the clean-room facility. The Schedule M guidelines issued by the Central Drugs and Standards Control Organization (CDSCO), Govt. of India, were adhered to wherever applicable.

### 2.3. Generation of Immature Dendritic Cells (IDCs) from Monocytes

Apheresis was performed without giving external cytokine stimulation. Around 50 mL of the product was used for mononuclear cell isolation using Ficoll^®^ Paque Premium (GMP-grade, density: 1.073 g/mL), (GE Healthcare, Chalfont St Giles, UK) as per the manufacturer’s instructions. After 30 min of centrifugation, plasma and the enriched fraction of mononuclear cells were collected and washed with DPBS (GMP-grade, Lonza, Basel, Switzerland) twice at 1580 rpm for 10 min at 15 °C. The cells were counted and injected at a concentration of 4 × 10^6^ cells/mL into cell culture bags (VueLife^® ^ “AC” series bags, Saint Gobain, Gaithersburg, MD, USA) and incubated for 30 min at 37 °C. After incubation, the non-adherent cells were withdrawn and the bag was washed with 10 mL of ice-cold DPBS twice. The VueLife^®^ A/C bags, which utilized a treated form of fluorinated ethylene propylene (FEP), enabled monocyte enrichment. The adherent cell fraction was differentiated to immature DCs (IDCs) in the presence of 600 IU/mL of granulocyte macrophage colony-stimulating factor (GM-CSF) and 50 IU /mL of interleukin 4 (IL4) in a CellGro^®^ medium for four days at 37 °C/5% CO_2_.

### 2.4. Validating Bag-Cultured Immature DCs for Antigen Uptake

On the 4th day of culture, around 1 × 10^5^ IDCs were withdrawn from the bags, washed and incubated for 30 min at 5% CO_2_ with FITC-Dextran (0.5 mg concentration), as described previously [19], and analyzed via flow cytometry (Moflo-XDP, Beckman Coulter Inc., Brea, CA, USA). Additionally, the IDCs (5 × 10^5^ cells/100 µL) were stained with DAPI and visualized on a fluorescent microscope (Leica, Wetzlar, Germany) at 40× magnification. The images were analyzed using the Image J software v 1.8.

### 2.5. Preparation of Antigenic Sources for DC Maturation

For the autologous tumor lysate preparation, a cervical punch biopsy was collected in 10 mL of Hank’s balanced salt solution (Lonza, Switzerland) (ice cold) containing 100 IU per mLs of penicillin, streptomycin, and gentamicin (Lonza, Switzerland) each. Sample processing was carried out on the same day of collection and processed in 6 mL of HBSS containing 0.6 Pz units of collagenase (NB6 GMP-grade from Serva GmBH, Heidelberg, Germany) and incubated overnight at 37 °C/5% CO_2_ to allow for tissue dissociation). Traces of collagenase were removed by washing with calcium-free DPBS. The final cell suspension was passed through a sterile 70 μm cell strainer (Miltenyi MACS, Waltham, MA, USA) to obtain a fine single-cell suspension. Approximately 2 × 10^5^ cells were immobilized on slides and verified by a cytologist to obtain the tumor cell percentage. The remaining cells in the single-cell suspension (SCS) were frozen in a ready-to-use, serum-free, GMP-grade cryopreservation medium with 10% Dimethyl sulfoxide (DMSO) (CTS™ Synth-a-Freeze™ Medium—Gibco™, Waltham, MA, USA) as per the manufacturer’s instructions. The tumor lysates were prepared as described previously [19] and evaluated for sterility followed by cryopreservation at −80 °C until use for antigen priming.

### 2.6. Dendritic Cell Maturation Using Two Different Antigenic Sources

On day 4, nonadherent IDCs were removed and counted. The whole-tumor lysate was added at a ratio of 3 tumor cells/1 DC and incubated for 2 h at 37 °C. Alternately, for rSPDC generation, GMP-grade rhSPAG9 protein (84 kDa), trademark SPAGNII™ (manufactured by Syngene International Pvt. Ltd., Bangalore, India; SPAGNII™), was used. The purified protein (dissolved in 150 mM of NaCl) vials had a concentration of 16 μg/vial and were stored at −80 °C. A previous validation of the rhSPAG9 antigen for maturation found 750 ng per million of immature dendritic cells to be optimal [3]. Subsequent to priming, the culture bags were incubated for two hours at 37 °C. After two hours, a proinflammatory cytokine cocktail consisting of TNF-α (100 IU/mL) and IL-1ꞵ (100 IU/mL) was prepared in a Cell Gro ^®^ DC medium and injected into to the culture bags for maturation until day 7.

### 2.7. Cryopreservation and Storage of MDCs

Following 7 days of culture, around 1.5 × 10^6^ mature DCs/mL were suspended in a GMP-grade CTS Synth-A-freeze medium (Gibco, Waltham, MA, USA) and stored in 12–20 cryovials. The effects of cryopreservation on stability and viability were analyzed via the random sampling of the cryopreserved PBMCs, IDCs (n = 5 each), and MDCs (TLPDCs: n = 3, rSPDCs: n = 2). The samples were analyzed at short (up to 3 h post-thaw) and long intervals following cryopreservation (from 15 days to 3 years).

### 2.8. Quality Control (QC) Measures for Large-Scale Dendritic Cell Production (Table 1)

QC criteria (shown in Table 1) were used to assess the precursor cell population as well as dendritic cell differentiation.

### 2.9. Phenotypic Expression

Around one million MDCs generated following 7 days of culture were washed and suspended in the plain Cell Gro DC^®^ medium for 24–48 h. Adherence and changes in morphology following cytokine withdrawal were monitored microscopically, and counts were obtained through trypan blue exclusion. All the QC assays were performed in MDCs recovered after the washout assay. For the phenotypic analysis, 2 × 10^5^ monocytes and immature and mature DCs were stained with the panel of antibodies along with isotype controls (Beckman Coulter Inc., CA, USA) as described in Table 1. The migratory capacity of TLPDCs and rSPDCs towards macrophage inflammatory protein-3 beta (MIP-3β: 300 ng/mL) and 6Ckine (R&D systems, Minneapolis, MN, USA, 250 ng/mL) was assessed in a 24-well plate using transwell inserts (Corning® Costar®, Corning, NY, USA) as carried out previously [17].

#### Differentiation and Maturation Index Calculation

Based on our previous studies using plate-generated DCs, the QC criteria were framed for several characteristics, including phenotypic expression and functional characteristics, that reflected monocyte-to-immature DC (IDC) differentiation as well as the maturation of IDCs to MDCs. Process-dependent-responder independent markers were first identified and utilized for calculating the individual differentiation and maturation scores of each batch.

For the differentiation scores (DSs) of the immature DCs, the percentages of phenotypic expression of the markers CD 80, 86, HLA-DR, CD40, and CD83 were considered, along with percentage of FITC dextran uptake, while the phenotypic expression of the same markers and the percentage of migration were used for calculating the maturation scores (MSs) for the MDCs following the flow cytometric analysis. The CD14 expression was downregulated and hence excluded from the calculation. For IDCs, wherever a range was specified for cut-off, the maximum value was taken for calculating the DS. The maximum percentage of cells (100%) expressing each marker or characteristic was considered as 1 (Table 1). We employed the cut-off to arrive at a cumulative DS of 3 for the IDCs and a cumulative MS of 3.95 for the MDCs as the base for calculating the differentiation index (DI) and maturation index (MI), respectively.

DI = DS of individual batch/3

MI = MS of individual batch/3.95

### 2.10. Proliferation Assay

Allogenic PBMCs (n = 36) were isolated from donor blood after density gradient separation with Ficoll^®^ Hypaque and subjected to plastic adherence to remove the monocytes. The non-adherent cells alone were stained with Carboxyfluorescein succinimidyl ester (CFSE) (Invitrogen, Carlsbad, CA, USA) and cultured with the irradiated MDCs (TLPDCs n = 18, RSPDCs n = 18) at a 1:50 ratio, as carried out previously [19]. The co-cultured cells were finally stained with anti-CD4 and -CD8 antibodies conjugated to PC5 and APC (IgG1, both from Beckman Coulter), respectively, along with a dead cell exclusion dye (LIVE/DEAD™—Thermo Fisher Scientific, Waltham, MA, USA) for 15 min, and fixed for analysis to verify the response of allogenic mononuclear cells (MNCs) upon stimulation with MDCs. 

### 2.11. Flow Cytometric Detection of IFNγ Secretion in Allogenic Responders

Non-adherent allogenic PBMCs were analyzed for intracellular secretion of interferon-gamma (IFNꝩ) after stimulation. These stimuli included TLPDC or rhSPAG9 DCs (1:50 ratio of DCs/PBMCs) or PMA (10 ng) and ionomycin (100 ng) as the positive control. The production of IFNγ was analyzed after 72 h of incubation in the Cell Gro medium^®^ in the presence of 10 mg/mL of brefeldin A (Sigma, MA, USA). In all samples, incubation was performed at 37 °C in a 5% CO_2_ incubator. The cells were washed, fixed, and subsequently stained for surface markers such as anti-CD4 PC5, anti-CD8 APC, and an intracellular marker anti-IFNꝩ FITC (all antibodies were IgG1, from Beckman Coulter Inc., CA, USA).

### 2.12. Microbiological Sterility and Assessment of Bacterial Endotoxin Level

Sterility tests, including direct inoculation, Gram-stain, and endotoxin assays, were carried out independently by the Department of Microbiology at the Cancer Institute. The detection of endotoxin lipopolysaccharide (LPS), the membrane component of Gram-negative bacteria, was performed using a Pierce^TM^ chromogenic endotoxin quantitation kit (Thermo Fisher Scientific, Waltham, MA, USA). A endpoint chromogenic limulus amoebocyte lysate (LAL) assay was performed according to the manufacturer’s guidelines with a detection range of 0.01–1.0 EU/mL. The color developed was read at 405 nm in a microplate reader (Thermo Fisher Scientific, Waltham, MA, USA). The concentration of endotoxin was calculated using a standard curve.

### 2.13. Statistical Analysis

The numerical data obtained from each experiment are expressed as mean ± SD unless otherwise indicated; the data were analyzed using Graph pad prism v 7.04. Student’s *t*-test was used for comparison and a *p*-value of less than 0.05 was considered statistically significant.

## 3. Results

### 3.1. Screening of Patients for HPV and SPAG9 Positivity

The clinicopathological parameters of the patients recruited for this study, including their HPV status (Figure S1a) and SPAG9 positivity, are shown in Table 2. The percentage of tumor cells positive for SPAG9 was assessed, and a cut-off of >20% was designated based on SPAG9 expression in the normal cervix tissue (n = 5) (Figure S1b). A score of zero to four was assigned to the tissues based on the percentage positivity and intensity, as described by Remmele W, Stegner HE et al. [20], to arrive at the IRS scores mentioned in the Table 2.

### 3.2. Monocyte Enrichment and Adherence Was Improved by Using Culture Bags with Coated Surfaces

**Figure 1:**
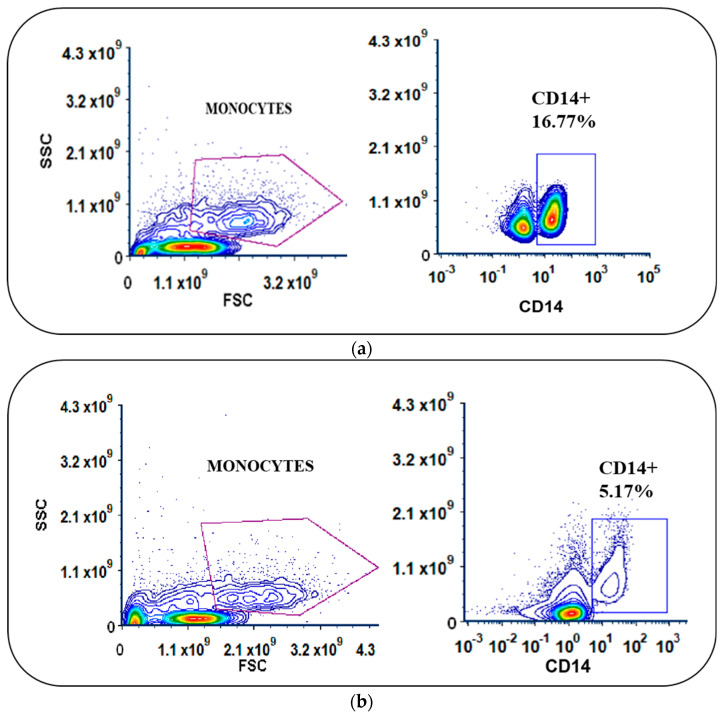

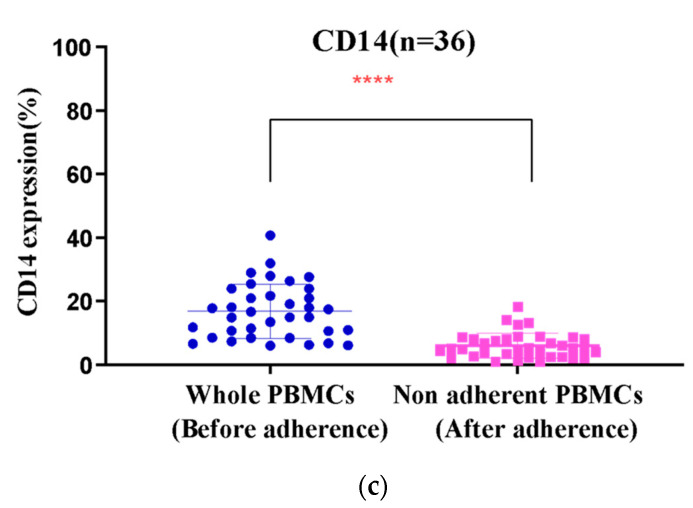
(**a**,**b**) Scatter plots of PBMCs with differences in the expression levels of CD14 in monocytes before and after adherence in cell culture bags. (**c**) Significant difference in percentage of CD14 expression (**** *p*  <  0.001) in monocytes.

Following apheresis, we performed density gradient centrifugation using Ficoll Paque Premium (1.073 g/mL), which is ideal for the isolation of low-density mononuclear cells such as monocytes. The average yield of total PBMCs was 1.1 × 10^9^ (±0.2) cells. Among the isolated PBMCs, an average of 19.13% were identified as monocytes (CD14+) in the flow cytometric analysis. The PBMCs were enriched for monocytes via adherence within the VueLife A/C culture bags. A significant difference in the percentage of CD14-expressing cells before and after medium withdrawal (Figure 1a–c) was observed (*p* < 0.0001), providing the data on the enrichment of monocytes. While analyzing the expression of CD14 in the non-adherent cells after incubation and washing, on average, we found that only around 7.2% (±8.2) expressed CD14, implying that nearly 92.02% of the initial monocyte population had adhered. The non-adherent cells that were aspirated out from the bag were counted and stored. A manual cell count using trypan blue exclusion showed that the average yield of non-adherent cells was 51 ± 6% after injection and withdrawal. The differentiation of enriched monocytes to IDCs was subsequently carried out with GM-CSF and IL-4 for 4 days.

On average, the percentage of adhered monocytes based on CD14 expression was 11.6 (±9.2%) (Figure 1b). In IDCs, this was reduced to 2.8% (±2.4), clearly indicating differentiation (Figure 1c). Using coated FEP bags, around 95 ± 4% of adherent monocytes differentiated into IDCs upon GM-CSF and IL4 treatment.

### 3.3. Bag-Cultured Immature DCs Showed Superior Antigen Engulfment Potential

**Figure 2:**
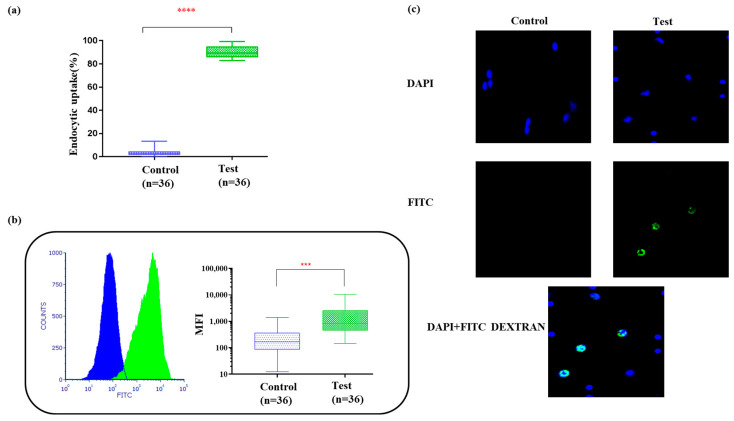
(**a**) Box plots show percentage of FITC dextran uptake (**** *p*  <  0.001). (**b**) Histogram and box plot for mean fluorescence intensity of the engulfed FITCs within 30 min of incubation at 37 °C/5% CO_2_. Paired *t*-test showed high significance (*** *p*  <  0.001). (**c**) Endocytic uptake of FITC dextran by IDCs visualized under 40× magnification. DAPI nuclear counterstaining was carried out and analyzed using Image J software.

IDCs have the capacity to engulf antigens and, hence, the endocytic uptake of FITC-dextran by IDCs is shown in Figure 2a. The median fluorescence intensity (MFI) of the test cells was 12-fold higher than that of the control cells (Figure 2b). The percentage of uptake in the test cells (90.26% ± 0.7) was also significantly higher than that of the controls (3.5% ± 0.4) (*p <* 0.001). A DAPI nuclear counterstain was used to determine the gross cell morphology (shown in Figure 2c). When we compared the results from the plate-cultured DCs reported previously [19], we found that the difference in MFI observed in the plate-cultured DCs was half (six-fold) of what was observed in the bag-cultured DCs. This showed the superiority of IDCs cultured in bags, which had superior gas exchange and dual surfaces for adhesion, enabling the uniform availability of cytokines to the cells for differentiation. Following the assessment of endocytic uptake, tumor antigens, either as whole-tumor lysates or rhSPAG9, were introduced on day 4 of culture. The mean tumor cell percentage following collagenase digestion was found to be 72% ± 3.2 (arm II, n = 18) in the samples obtained. Our method was found to be optimal for yielding sterile tumor cells, devoid of microbial contamination from cervical tumor punch biopsy samples, and priming was carried out at a 3:1 tumor cells/DCs ratio based on our previous studies with plate DCs and small culture volumes. rhSPAG9 protein at a concentration of 750 ng/mL/million cells was also optimized in our earlier study [3]. Since rhSPAG9 was synthesized commercially under GMP, the same was used for priming up to 15 million IDCs (n = 18).

### 3.4. Bag Generation Minimized DC Loss to Adherence and Maintained DC Phenotypes despite Cytokine Withdrawal

Mature DC loss due to adherence to culture plates after priming was previously observed. Such adherence would severely hamper the batch production process. Hence, this phenomenon was monitored closely during the maturation process. We found that around 15% of TLPDCs (±2) and 10% of rSPDCs (±3) were lost due to adherence during maturation. Overall, for both matured DCs (n = 18 each), the yield at maturation was approximately 50–70% of the input IDCs, implying that there could be a 30 to 50% loss due to adherence. The reason for this was not understood in our current study.

#### Phenotypic Expression of Batch-Produced DCs

The phenotypic expression analysis showed that, consistently, in all the MDCs, (irrespective of the antigenic priming source), the expression of maturation marker CD83 showed an average six-fold increase, which was found to be highly significant (IDC vs. MDCs: *p* < 0.001) in bag-cultured DCs compared to the plate-cultured DCs (*p* = 0.02) in our previous studies.

**Figure 3:**
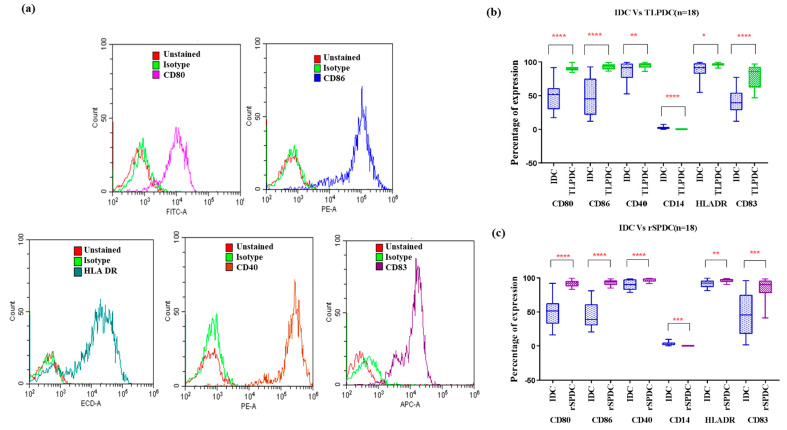
(**a**) Representative histograms for each phenotypic marker expression analyzed using flow cytometry. (**b**) IDCs vs. TLPDCs (n = 18); (**c**): IDCs vs. rSPDCs (n = 18). Box plots for the difference in expression of phenotypic markers in IDCs and MDCs (TLPDCs (**b**) and rSPDCs (**c**)). Paired *t*-test: * *p*  <  0.05, ** *p*  <  0.01, *** *p*  <  0.001, **** *p*  <  0.001.

Other co-stimulatory markers, such as CD80 and CD86, were also increased by more than two-fold in comparison to IDCs. Both CD40 and HLA-DR expression was high in IDCs and remained at similar levels in MDCs. While IDCs were observed to retain a slightly higher expression of the precursor monocyte marker CD14 (2.8% ± 0.32, n = 36), it was found to be decreased in MDCs (TLPDCs: 0.3% ± 0.04, rSPDCs: 0.2% ± 0.03) (Figure 3b,c).

### 3.5. Antigen-Primed Bag-Cultured DCs Migrate Efficiently and Stimulate Allogeneic T Cells to Proliferate

**Figure 4:**
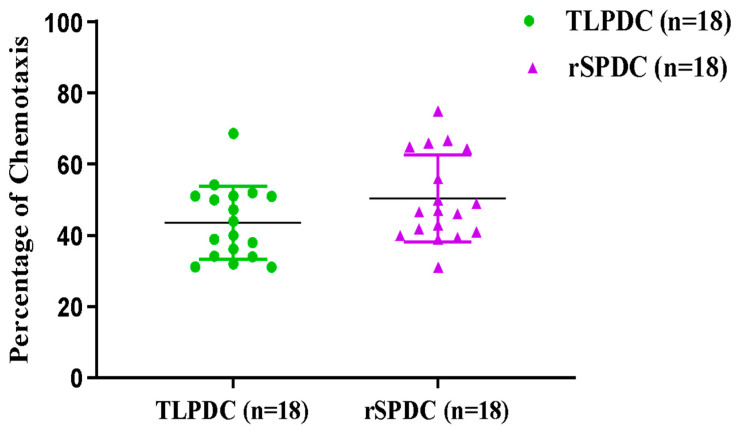
Analysis of DC migration: scatter plot showing >30% of chemotaxis in TLPDCs and rSPDCs. The number of DCs migrating towards CCL19 and CCL21 in the transwell inserts was identified using flow cytometry.

A notable chemotactic response was observed in bag-cultured MDCs (both rSPDCs and TLPDCs) towards CCL19 and CCL21. Both chemokines induced almost a five-fold increase in the migratory response of DCs compared with the negative controls (passive diffusion control), and a significant increase was also found in MDCs compared to IDCs (paired *t*-test, *p* < 0.0002). These results indicated that MDCs (both TLPDCs and rSPDCs) exhibited strong chemotaxis (Figure 4). Within three hours, around 43.6% of TLPDCs and 50.3% of rSPDCs had migrated across the transwell inserts to the chamber below, in response to the chemokine pair.

**Figure 5:**
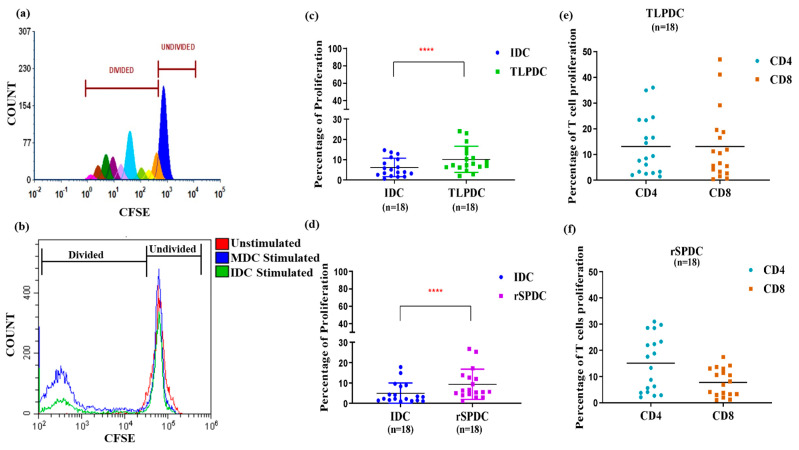
CFSE dye-based proliferation assay. (**a**) Representative histogram showing different generations of divided populations. (**b**) Histogram showing overlay of CFSE intensity in allogenic PBMCs stimulated by IDCs and MDCs at 1:50 DC/PBMC ratio. Scatter plot for overall proliferation of PBMCs upon stimulation with IDCs and MDCs (TLPDCs (**c**) rSPDCs (**d**)). Paired *t*-test for IDCs and MDCs showed significance (**** *p*  <  0.001). Percentage of CD4 and CD8 proliferation is represented in (**e**,**f**).

Apart from migration, the functional efficacy of the bag-generated DCs was evaluated based on their ability to stimulate proliferation in allogeneic donor PBMCs (Figure 5a). IDCs, which were used as internal controls, induced only minimal proliferation (Figure 5b). In a paired comparison of IDCs vs. TLPDCs and IDCs vs. rSPDCs, there was a 1.7- and 1.8-fold better proliferation response, respectively. These results were highly significant (*p* < 0.0001) (Figure 5a,b). When the percentages of CD4 and CD8 cells within the proliferated cell populations were analyzed, there was once again a significant increase in the percentage of both CD4+ helper T cells and CD8+ cytotoxic T cells with respect to both unstimulated (*p* < 0.001) and IDC-stimulated controls (*p* < 0.001) (Figure 5c,d). It is noteworthy that with TLPDCs, a higher CD8+ T cell response was observed (TLPDCs: 13 ± 3.2% vs. rSPDCs: 8 ± 1.5%) (Figure 5e) while CD4+ T cell proliferation was slightly higher in the rSPDC-stimulated co-cultures (TLPDCs: 14 ± 3.2%; rSPDCs: 15 ± 2.5%) (Figure 5f); however, these differences were not statistically significant.

### 3.6. Evaluation of Inter-Batch Consistency

**Figure 6:**
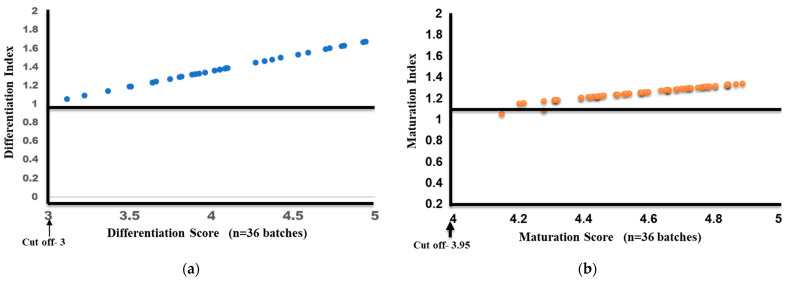
Differentiation index and score (**a**) and maturation index and score (**b**) for 36 batches of MDCs.

Based on our previous studies using plate-generated DCs, the QC criteria were framed for several characteristics, including the phenotypic expression of DC markers and functional characteristics, that reflected the monocyte-to-IDC differentiation as well as maturation. For IDCs, the phenotypic expression and FITC dextran uptake on day 4, prior to maturation, were used to obtain the differentiation score (DS) since both of these characteristics were evaluated using flow cytometry. Using the percentage of cells expressing each marker based on the cut-off (Table 1), with the maximum taken as one (100%), we arrived at a cut-off differentiation score of two for IDCs. CD14 alone, which is downregulated during differentiation, was excluded from the calculation. Similarly, for mature DCs, the percentage of migration, also evaluated using flow cytometry, was included instead of endocytic uptake, and using the average plate DC values as the baseline, we arrived at a cut-off score of 3.95 as the maturation score (MS). Using these values, a differentiation (DI) and maturation index (index = DS/MS of each batch/DS/MS cut-off) were calculated for each individual patient’s batch (n = 36). The IDCs had a DS ranging from 3.1 to 5.1 (n = 36) with an average DI of 1.39 (±0.171), clearly indicating differentiation. Similarly, the batch-produced MDCs had a median maturation score of 4.59 (range: 4.15–4.88), while the average MI was 1.251 (SD ± 0.062). Figure 6a,b show the variation in differentiation and maturation for all 36 batches. Both figures clearly show that maximum variation could be seen in the DI, which is also dependent on the percentage of monocytes in the input samples. However, the MI clearly shows that, post-differentiation, maturation could be synchronized with very little variation following our seven-day protocol for consistent batch production.

### 3.7. Assessment of Microbiological Sterility

Although housed within sterile culture bags with a facilitated gas exchange within a CO2 incubator, the sterility of the final product was ensured by subjecting the spent medium to a series of microbiological tests, routinely performed in the Dept of Microbiology at CI (WIA). Additionally, the LAL test was performed to detect and quantify Gram-negative bacterial endotoxins in the MDC culture medium of all batches. The level of endotoxins in the spent DC culture medium was found to be 0.5 ± 0.08 EU/mL. Fungal contamination was ruled out by monitoring the cultures for up to 96 h post-production. All 36 batch cultures from individual patients, spanning a length of three years from January 2017 to December 2019, were consistently found to be sterile, indicating that our scaled-up, minimally automated method could yield mature DCs with sterility and reproducibility.

### 3.8. MDCs Generated on a Large Scale Have the Capacity to Stimulate the Secretion of IFNγ in Allogenic PBMCs

**Figure 7:**
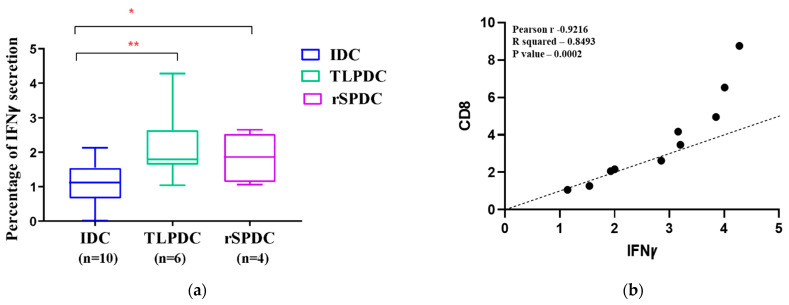
(**a**) Box plot showing IFNꝩ secretion by PBMCs upon stimulation with IDCs and MDCs. A significant difference was seen between IDCs and MDCs (** *p*  <  0.01 and * *p*  <  0.03). (**b**) Correlation between percentage of proliferated CD8 cells and IFNꝩ secretion.

DCs generated using the manual method were evaluated for their ability to induce a Th1 response mediated by IFNγ after three days of DC/PBMC co-culture. Upon analysis, our results revealed that out of 36 patients, only 10 patients exhibited a strong response within 72 h of stimulation when detected using flow cytometry. Of these responders, six were TLPDC-induced cultures, which showed a two-fold higher proliferation when compared to the internal IDC controls (*p* < 0.03), while four of the responders were rSPDC-induced cultures, which showed a 1.2-fold higher response (*p* = 0.05). These results clearly show that both TLPDCs and rSPDCs were capable of inducing IFNꝩ secretion in effector T cells (Figure 7a). The statistical analysis showed that IFNꝩ secretion had a significant correlation with an increase in CD8 T cell proliferation in both TLPDC and rSPDC cultures (n = 10, *p* = 0.0002, Pearson r = 0.9216) (Figure 7b). Hence, a pro-inflammatory Th1 response could be induced using DCs matured with both types of antigenic sources.

### 3.9. Consistency in Bag Generation Ensures Stability during Short- and Long-Term Cryopreservation

**Figure 8:**
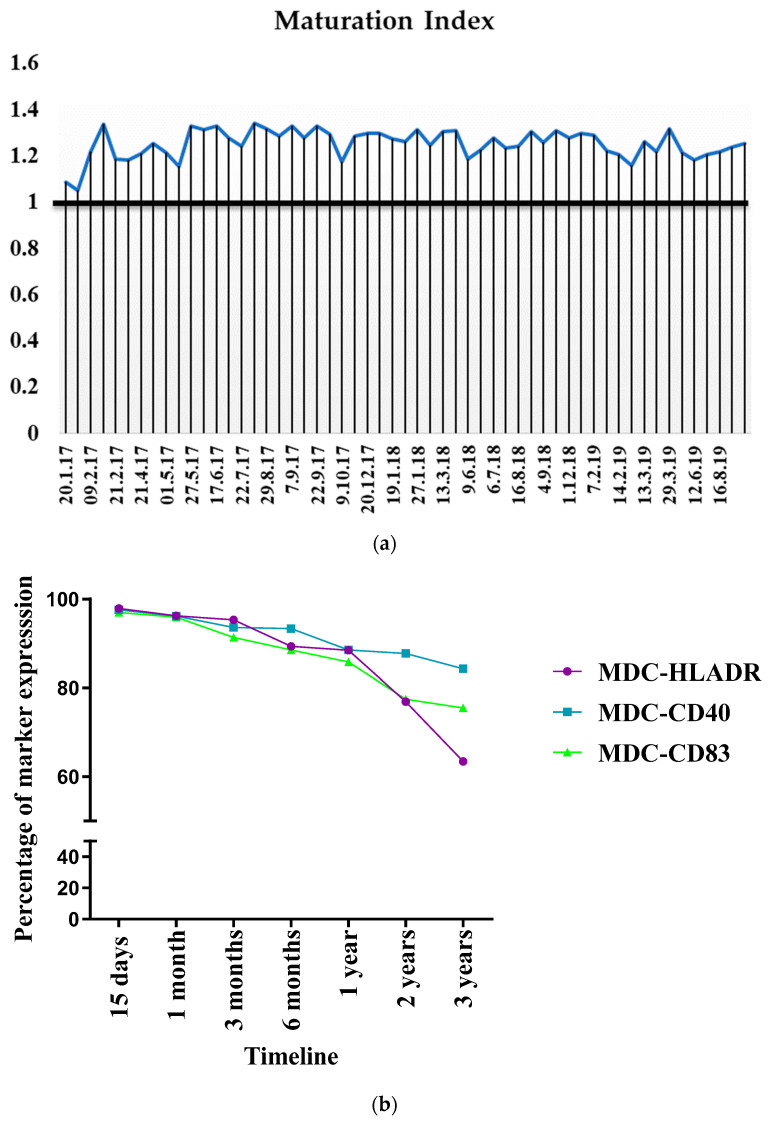
(**a**) Consistency in DC maturation. (**b**) Stability of DCs after cryopreservation and expression trend of immunophenotypic markers at various time intervals after cryopreservation. At each indicated time point, cells were subject to rapid thawing at 37 °C, washed, and stained with anti-HLA DR-ECD, anti-CD40 PE, and anti-CD83 APC, and analyzed via flow cytometry.

The inter-batch consistency of mature DC generation was evaluated over a period of 32 months from January 2017 to July 2019. Since mature DCs were cryopreserved and used for vaccination, the batch mode generation efficacy was evaluated with the MI for an inter-batch comparison. Figure 8a shows that consistency was achieved during maturation despite a higher spread in the DI. The generation was handled by three different personnel and there was no significant difference witnessed between the batches over the three years.implying that process optimization could be achieved with the protocol used.

Random sampling was then carried out to study the effect of cryopreservation on cellular viability, yield, and surface marker expression after short- and long-term cryopreservation. Shorter time intervals (0, 30 mins, 1 h, 2 h, 3 h) allowed us to check the stability of cells before vaccination to determine the maximum interval between vaccine preparation and administration that could be allowed to account for contingencies. Table 3 shows that both types of MDC, suspended in physiological saline before the administration, were viable. Beyond half an hour, the cells could be stored at 2–8 °C for up to three hours without compromising their viability.

Upon retrieval from cryopreservation, the viability of the MDCs was largely unaffected. With the scaled-up process of DC generation and maturation, >90% of MDC viability was observed up to three years after cryopreservation. The phenotypic expression of the cryopreserved cells was tracked for up to three years, which showed that the expression of the markers did not show significant downregulation for up to two years after cryopreservation. However, after two years and three years, HLA DR showed a 1.2- and 1.5-fold reduced expression, respectively, while CD83 showed a 1.2-fold reduced expression after two years of cryopreservation (Figure 8b).

### 3.10. Establishment of Standardized Workflow and in-Process QC for Product Release

**Figure 9:**
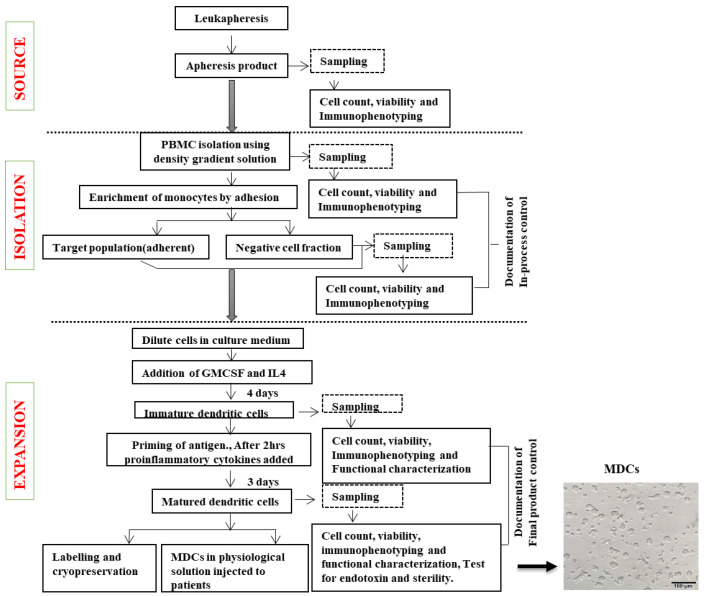
Steps involved in DC generation. Flow chart of the process of DC generation depicting blood collection, cell separation, cell culture, and quality control.

Based on our validation studies and the results for each of the batch-generated DCs, the detailed workflow for DC generation was established, as shown in Figure 9. Based on this protocol, we were able to ensure that training could be handled easily and reduce batch-to-batch variations. The optimization of the QC criteria ensured that the batch-produced DCs met the necessary cut-off limits for achieving clinical use status.

## 4. Discussion

In the current study, we standardized large-scale DC generation by performing a two-step monocyte enrichment using Ficoll Paque Premium of a density of 1.073 g/mL, followed by adherence to specialized FEP bags possessing a higher surface energy. Although several techniques of monocyte isolation, such as magnetic or mechanical separation, exist, and automated features for monocyte enrichment have been validated for GMP compliance [21,22], being an academic CRC attached to a voluntary charitable hospital, resource constraints prompted the adaptation of an economically sustainable enrichment method without compromising on GMP. In a study conducted by Figueroa et al., there was no significant difference in the yield and viability of monocytes isolated by CD14 magnetic separation and plastic adherence [23]. This method was straightforward with minimal damage to cells, and did not require complex processing steps. However, the purity was compromised [24]. Nevertheless, this method did not affect the quality or functionality of the dendritic cells and assured the safety of the final product. Another method of monocyte enrichment is using the counterflow centrifugal elutriation (CCE) method, which could produce a 80% recovery of monocytes, albeit requiring expensive processing equipment and trained operators [25].

Several culture vessels have been evaluated for their suitability to generate large-scale DCs with materials like polystyrene (culture flasks), polyolefin (bags), and FEP (bags) [26,27]. Such studies showed that cell culture bags like the FEP-based VueLife A/C, which had a higher surface energy, were ideal, as they could easily accommodate clinical-scale volumes of starting and in-process material and were superior to flask-cultured cells in terms of yield, viability, and function with a reduced risk of contamination [28,29]. Our own results showed that a closed FEP-based system had the advantage of twin surface adherence for monocyte enrichment. Although previous studies, including ours, had recommended 1 million cells/mL for plate culture, optimization of the cell density (cells/mL) was performed to arrive at 300 million whole PBMCs for efficient adherence. With coated bags, a 73% (±4) adherence was observed, while with plates, we previously observed a 60% monocyte adherence [3,19].

Reports on phenotypic differences between flask- and bag-cultured DCs have been contradictory when polyolefin bags were used [30,31]. However, no such difference was noted between flask- and FEP-generated DCs [32]. Our results also showed no phenotypic difference when we scaled up from plates to cell culture bags. Since immature DCs can induce regulatory T cells, causing a risk of immune tolerance [33,34], the generation of homogeneous, mature, and functional DCs is critical for cancer immunotherapy. Various protocols for differentiation and maturation have been developed over the years. Although a short-term culture protocol may be good at minimizing the risk of contamination, saving labor, cost, and time for large-scale clinical use, such fast DCs were found to be less resistant to IL-10 induction with certain maturation cocktails [35]. Since our previous in vitro studies showed a significant difference in antigen uptake among IDCs between day 2 and day 4, we induced DC maturation on day 4. In line with our protocol, other groups also found the eight-day protocol to be optimal for DC generation when compared to a 10-day protocol [36,37]. Our previous results with plate-cultured DCs showed that close to 85% of immature DCs were capable of antigen uptake by day 4 [19]. In comparison, this was also improved to around 91% with bag-cultured DCs in the scaled-up protocol.

Although crude lysate preparations as a whole have been used previously for the small-scale generation and maturation of DCs, only a few studies have reported their use in clinical trials in the form of primary cell cultures while scaling up the process [38]. Given the poor success rates of primary cultures in general [39], the preparation of tumor lysates may be a better alternative. DC vaccines matured with whole-tumor lysates for glioma used a fixed protein concentration after lysate preparation for priming DCs [37]. Although similar, our approach is unique in that, based on previous in vitro studies, we carried out priming at a tumor cell/DC ratio of 3:1. The purification of tumor cells to minimize the presence of infiltrating or stromal cells without compromising on sterility could not be achieved. Hence, crude, whole-tumor lysates could be efficiently scaled up without compromising on the antigen content by keeping this ratio constant while priming. Although several methods such as transfection and electroporation for antigen loading exist [40], the natural endocytic route of antigen capture may be the least compromising and less expensive, requiring no additional equipment.

DC vaccine release criteria have been proposed by the iSBTc−SITC/FDA/NCI Workshop on Immunotherapy Biomarkers [41], which requires a minimum of 70% viability with MHC class II and CD86 expression in at least 70% of the cells. The additional characterization of proteins expressed by DCs including MHC class I, CD80, CD83, and CCR7 is required for clinical application. Hence, our QC criteria were framed based on SITC recommendations as well as results from our previous studies, considering the aspects of viability and purity (phenotypic expression). While measuring potency, our own studies showed that plate-generated DCs exhibited a wide variation in migratory capacity, while the bag-generated DCs in the current study showed more consistency. Nevertheless, our previous studies showed that CCR7, which is the receptor for both CCL19 and 21, was elevated upon maturation [3]. We plan to incorporate CCR7 as the surrogate for migratory analysis in future studies. Another limitation of the study is the use of an intracellular IFNꝩ flow cytometry assay. Although it is a direct-potency assay with a possibility to phenotype simultaneously antigen-reactive T cells, intracellular IFNꝩ detection via flow cytometry in PBMCs mandates the longer incubation of antigenic stimulation in vitro to achieve a measurable response [42]. Hence, this was not used as a product release criterion under QC.

Although cryopreservation affects cell viability drastically, our results showed that DC viability was close to 90% even after 3 years. Similarly, Celluzzi et al. observed cell viability of approximately 82% after nearly 24 months of storage in 5% DMSO with a cryoprotectant like hydroxyethyl starch (HES) [43]. More recently, Kawaguchi et al. showed long-term survival when DCs were generated in clusters and cryopreserved [44]. Pardali et al. observed that Synth-a-freeze could be used for cryopreserving monocytes, which did not affect their migratory capacity and viability upon short-term cryopreservation [45]. A similar study in CML patients showed no change in DC phenotypic expression due to cryopreservation for a short duration [46]. Our results also showed, using the same cryoprotectant, that the viability of IDCs and MDCs was unaffected. Interestingly both IDCs and MDCs possessed phenotypic stability for up to one-year; beyond this, there was a drop in the expression of HLA-DR and CD83. No other studies have so far reported stability data for DCs beyond three years. The final product release was carried out based on the cell count and viability through a trypan blue exclusion test. More than one million DCs and an 80% viability were mandatory for final product administration to patients.

## 5. Conclusions

This is the first study of its kind from India for cervical cancer. We present, in the current study, a manual, semi-automated process, optimized for generating dendritic cells of therapeutic grade. Our process required basic clean-room and equipment facilities, maintained to GMP standards without expensive automation. The harmonization process ensured that there was no variation during the manufacturing except for the priming step. Our QC criteria, which can be employed to test the stability and potency of both types of DCs, reflected the consistency of the generation process. Our method also showed very little operator-dependent process variation, ensuring standardized, reproducible therapeutic-grade DC generation.

## Figures and Tables

**Table 1:** Factors required for QC assessment in GMP-grade DC production.

Attributes (Method)	Assay	IDC (Max—100%, Differentiation Score = 1 *)	MDC (Max—100%, Maturation Score = 1 ^#^)
IDENTITY (Flow cytometry)	Immunophenotype	Cut-off	Cut-off
	anti-CD14 PC5 (IgG1 Mouse)	<5%	0–0.5%
	anti-HLA-DR ECD (IgG1 Mouse)	>80% (0.8) *	>90% (0.9) ^#^
	anti-CD40 PE (IgG1 Mouse)	>75% (0.75) *	>90% (0.9) ^#^
	anti-CD80 FITC (IgG1 Mouse)	>10% (0.1) *	>80% (0.8) ^#^
	anti-CD86 PE (IgG1 Mouse)	5–50% (0.5) *	>85% (0.85) ^#^
	anti-CD83 APC (IgG1 Rat)	0.5–5% (0.05) *	>20% (0.2) ^#^
POTENCY (Flow cytometry)	FITC dextran uptake	>80% (0.8) *	No data
	Migration towards CCL19/21 in 3 hrs time	None or <1%	>30% (0.3) ^#^
	Proliferation of allogeneic PBMC	Percentage of cell division (overall, CD4, CD8T cells)	Percentage of cell division (overall, CD4, CD8 T cells >IDCs)
STERILITY	Clinical tests before apheresis	Blood sample negative for HIV, HBV and HCV	
	Microbiological tests (direct inoculation and Gram stain)	Sterile upon test for bacterial and fungal culture up to 2 weeks	
	Test for endotoxin	Level of endotoxin less than 1.0 EU/ml	

* cut-off values used for differentiation score, # cut-off values used for calculating maturation score.

**Table 2:** Provides clinicopathological features along with HPV status and SPAG9 expression of all the screened patients (n = 182).

Clinicopathological Features of Screened Patients	No. of Patients
Patient classification:	
Median age in years (range)	52 (30–65)
Locally advanced (Stage III B)	146
Metastatic (Stage IV)	36
Cell type (WHO classification):	
Squamous cell carcinoma	176
Papillary squamous cell carcinoma	2
Adenocarcinoma	1
Adenosquamous carcinoma	0
Poorly differentiated carcinoma	3
Histological Grade:	
Grade 1	1
Grade 2	48
Grade 3	133
Keratinization status:	
LCNK	129
LCK	53
HPV status:	
HPV-Positive	181
HPV-Negative	1
IRS score for SPAG9:	
0—Negative	0
1 to 3—Mild positivity	33
4 to 8—Moderate positivity	119
9 to 12—Strong positivity	27
Tumor Percentage (range)	72 (50–90%)
Total number of patients recruited for trial	54/182

**Table 3:** Stability of a DC vaccine 0–3 h of Post-Thaw.

Duration	Percentage of Viability Post-Thaw *	
	TLPDCs (n = 5)	rSPDCs (n = 5)
0	97 ± 1.3	96 ± 2
30 min	97 ± 0.7	96.4 ± 00.8
1 h	95 ± 2	96 ± 1.2
2 h	95 ± 2	96 ± 1
3 h	94 ± 2	95 ± 2

* cells suspended in saline and stored at 2–8 °C.

## Data Availability

The data that support the findings of this study are contained within the article and Supplementary Material.

## Supplementary Materials

The following supporting information can be downloaded at https://www.mdpi.com/article/10.3390/vaccines12020112/s1, Supplementary Figure S1a: Agarose Gel electrophoresis image of GP5+ GP6+ PCR products of 120bp-representative tissue samples (T1 to T4) from lane 3 to 6. Supplementary Figure S1b: Immunohistochemical staining of SPAG9 in cervical cancer tumor tissue. Supplementary Table S1: Inclusion and exclusion criteria for patient selection.
