# Supplementary material for: Optimization and Validation of a Harmonized Protocol for Generating Therapeutic-Grade Dendritic Cells in a Randomized Phase II Clinical Trial, Using Two Varied Antigenic Sources
Source: Vaccines (Basel). 2024 Jan 23;12(2):112. doi: 10.3390/vaccines12020112 (PMC10892253; doi:10.3390/vaccines12020112)
Supplement: Supplementary file 1 [file vaccines-12-00112-s001.zip › vaccines-2713941-supplementary.pdf]

Optimization and Validation of a Harmonized Protocol for Generating Therapeutic-Grade Dendritic Cells in a Randomized Phase II Clinical Trial, using two varied antigenic sources.

Supplementary figures:

Supplementary Figure S1a

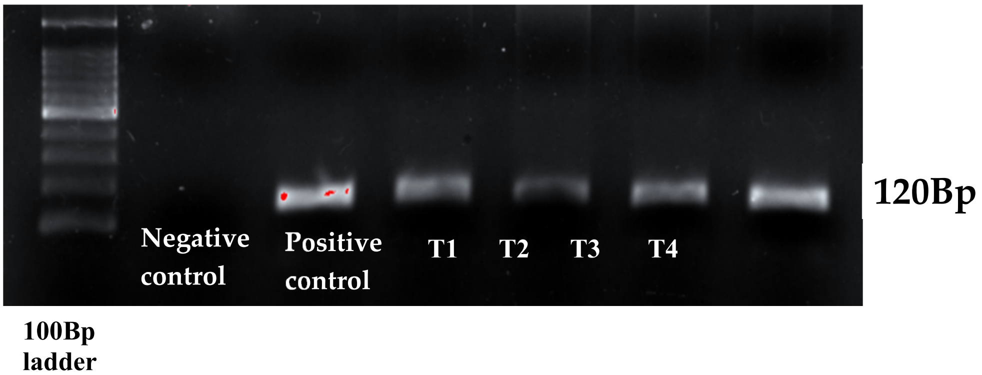

Supplementary Figure S1a: **Agarose Gel electrophoresis image of GP5+ GP6+ PCR products** of 120bp-representative tissue samples (T1 to T4) from lane 3 to 6

Supplementary Figure S1b

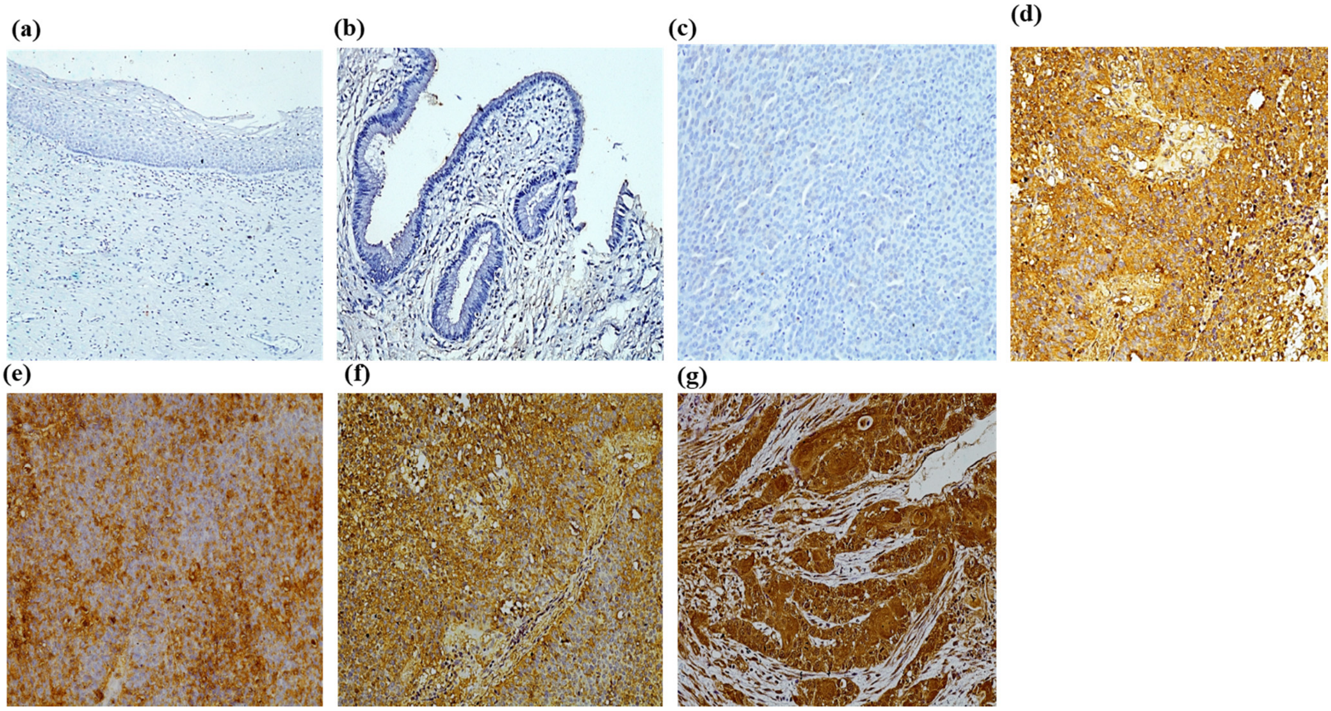

Supplementary Figure S1b: **Immunohistochemical staining of SPAG9 in cervical cancer tumor tissue.**

Weak staining of SPAG9 in non-cancer tissues (a) Ectocervix (Normal tissue); (b) Endocervix (Normal tissue); (c)Negative control; (d) Positive control (e) 50% of cervical squamous cell carcinoma tissue showing moderate

positivity for SPAG9 with 2+ intensity; (f) 80% of cervical squamous cell carcinoma tissue showing strong positivity for SPAG9 with 2+ intensity; (g) 90% of cervical squamous cell carcinoma tissue showing very strong positivity for SPAG9 with 3+ intensity ; magnification-20x.

Supplementary Table S1: Inclusion and exclusion criteria for patient selection

**Inclusion criteria**	**Exclusion criteria**
Subjects in the age group of 18-65	HIV or hepatitis B or C infection
Squamous cell carcinoma at Stage IIIB	Auto-immune disease and Acute infection
No prior radiotherapy or chemotherapy for any other malignancy	Pregnancy and Unwillingness to use a medically accepted form of birth control during the study
Karnofsky score of 70 or greater	Severe pulmonary or cardiac disease
No allergy to components of dendritic cells	Uncontrolled diabetes or hypertension
Hematological parameters<br>(LFT, RFT, SGOT, SGPT, and ALP)	Acute or chronic medical or psychiatric conditions
No contraindications for chemotherapy with cisplatin.	Patients on immunosuppressive drugs including steroids.
Evidence of a personally signed and dated informed consent document indicating that the subject (or a legally acceptable representative) has been informed of all pertinent aspects of the study	Significant obstructive uropathy
Subjects who are willing to comply with scheduled visits, treatment plans, laboratory tests, and other study procedures.	Patients who had participated in any other clinical trial during the past 90 days.
